# Cost-utility analysis of ^177^Lu-PSMA-617 radioligand therapy in second-line and third-line treatment for metastatic castration-resistant prostate cancer (mCRPC) in Germany

**DOI:** 10.1007/s00259-025-07317-9

**Published:** 2025-05-08

**Authors:** Carolin Brinkmann, Richard P. Baum, Tom Stargardt

**Affiliations:** 1 Universität Hamburg, Hamburg Center for Health Economics, Esplanade 36, 20354 Hamburg, Germany; 2 International Centers for Precision Oncology (ICPO), Wiesbaden, Germany; 3 CURANOSTICUM Wiesbaden-Frankfurt at DKD Helios Klinik, Wiesbaden, Germany

**Keywords:** Prostate cancer, Metastatic castration-resistant prostate cancer, Radioligand therapy, ^177^Lu-PSMA-617, Cost effectiveness, Cost utility

## Abstract

**Purpose:**

To evaluate the cost-effectiveness of ^177^Lu-PSMA-617 radioligand therapy (PRLT) in metastatic castration-resistant prostate cancer (mCRPC) in Germany by comparing (I) PRLT plus standard-of-care (SoC) versus SoC alone as third-line treatment and (II) PRLT versus second-line cabazitaxel chemotherapy.

**Methods:**

Cohort state-transition models were developed with (I) four health states (treatment, stable after treatment, progression, death) and (II) six health states (treatment, stable after treatment, third-line treatment after progression, stable after third-line treatment, next progression, death). Transition probabilities were derived from the VISION and TheraP trials, and quality-of-life data from the VISION and CARD trials. Costs were derived from statutory health insurance claims data (2019–2022). Models simulated a five-year horizon with one-month cycles, applying within-cycle correction and a 3% discount rate. Sensitivity analyses addressed uncertainty.

**Results:**

For Model I, PRLT plus SoC compared to SoC incurred incremental costs of €27,200 per patient, with a gain of 0.39 quality-adjusted life years (QALYs) and incremental cost-effectiveness ratio (ICER) of €69,418 per QALY gained. In Model II, PRLT plus SoC compared to cabazitaxel achieved incremental savings of €1,460 per patient and a QALY gain of 0.11, making it the dominant option.

**Conclusion:**

Although Germany lacks an explicit willingness-to-pay threshold for interpreting the ICER of Model I, it falls within the range of other reimbursed cancer therapies. This suggests PRLT is cost-effective as second- or third-line treatment for mCRPC. Between 2019 and 2022, hospitals mainly used self-produced ^177^Lu-PSMA-617, which was less costly than the product now commercially available, limiting the generalizability of our findings.

**Supplementary Information:**

The online version contains supplementary material available at 10.1007/s00259-025-07317-9.

## Background

Prostate cancer is one of the most frequently diagnosed malignancies in Europe [1] and is associated with a high burden of disease [2]. In cases where the disease progresses to metastatic castration-resistant prostate cancer (mCRPC), the burden increases further. According to European guidelines, recommended first-line treatment options for mCRPC are the androgen receptor pathway inhibitors abiraterone or enzalutamide, immunotherapy, or taxane-based chemotherapy with docetaxel [3]. Second-line options include cabazitaxel chemotherapy, a different androgen receptor pathway inhibitor, or Radium-223 for bone metastases [3].

A recently approved treatment option is Lutetium-177 vipivotide tetraxetan, which targets both visceral *and* bone metastases and has demonstrated an acceptable safety profile [4]. This therapy is indicated for patients with prostate-specific membrane antigen (PSMA)-positive mCRPC previously treated with an androgen receptor pathway inhibitor and taxane-based chemotherapy. The randomized, multicenter, international phase III VISION trial compared ^177^Lu-PSMA-617 radioligand therapy (PRLT) plus standard of care (SoC) to SoC alone^1^ in 813 PSMA-positive mCRPC patients [5]. Patients who received PRLT plus SoC experienced significantly prolonged progression-free survival (PFS) compared to SoC (median: 8.7 months vs. 3.4 months; hazard ratio for progression or death: 0.40) and overall survival (OS) (median: 15.3 months vs. 11.3 months; hazard ratio for death: 0.62) [5]. Similarly, the Australian randomized, multicenter phase II TheraP trial compared PRLT to cabazitaxel in 200 PSMA-positive patients. The trial found higher prostate-specific antigen response and delayed progression (hazard ratio: 0.63) with PRLT but no difference in OS between the study groups [6, 7].

PRLT offers certain advantages over SoC that are consistent with patient-reported treatment preferences. While OS appears to be the most important factor influencing treatment choice [8], preference studies in European mCRPC patients have identified controlling bone pain and delaying chemotherapy as additional priorities [9]. Thus, clinical research is increasingly evaluating the effects of PRLT in earlier treatment settings before chemotherapy, such as in hormone-sensitive [10, 11] or taxane-naïve patients [12, 13].

Treatments for mCRPC place a substantial economic burden on health systems worldwide [14, 15]. For example, in 2017, monthly all-cause health care costs based on claims data from statutory health insurers in Germany ranged from €959 for patients receiving best supportive care to €7,631 for those treated with cabazitaxel [16]. Assessing resource consumption alongside the clinical effects of new therapies compared to current practice is essential to ensure the efficient allocation of scarce health care resources. In cost-effectiveness analyses, the incremental costs between a new therapy and current practice are evaluated in relation to the corresponding incremental health benefit. This relation of incremental costs and incremental health effects is known as the incremental cost-effectiveness ratio (ICER). The health effect is frequently measured in quality-adjusted life years (QALYs), with one QALY representing one year in full health [17].

The cost-effectiveness of PRLT has been evaluated in several countries, primarily using data from the VISION trial [5]. In the United States, the ICER of PRLT plus SoC versus SoC alone was estimated at US$ 200,708 per QALY [18]. In Norway, PRLT plus SoC resulted in a QALY gain of 0.44 compared to SoC alone, but the ICER was not made publicly available [19]. In the United Kingdom, PRLT was not considered cost-effective compared to best supportive care and cabazitaxel; the ICER was redacted here as well [20]. In Australia, a preliminary analysis of ^177^Lu-PSMA-i&t (imaging and therapy) comparing its cost-effectiveness to a weighted combination of best supportive care and cabazitaxel, found it not to be cost-effective at an ICER of AU$ 93,947 per QALY [21]. A second analysis using updated evidence for modeling is currently ongoing [22]. To the best of our knowledge, the cost-effectiveness of PRLT has not yet been studied in the German health care context.

Our study therefore aimed to assess the cost-effectiveness of PRLT from the perspective of the German statutory health insurance system using real-world claims data. The analysis had two main objectives: first, to evaluate PRLT plus SoC versus SoC alone as a third-line mCRPC treatment (i.e., after two courses of chemotherapy), thus reflecting current treatment practice (Model I); and, second, to compare PRLT to second-line cabazitaxel chemotherapy to examine the cost-effectiveness of using PRLT earlier in the treatment sequence (i.e., after one course of chemotherapy) (Model II).

## Methods

### Model structure

**Fig. 1:**
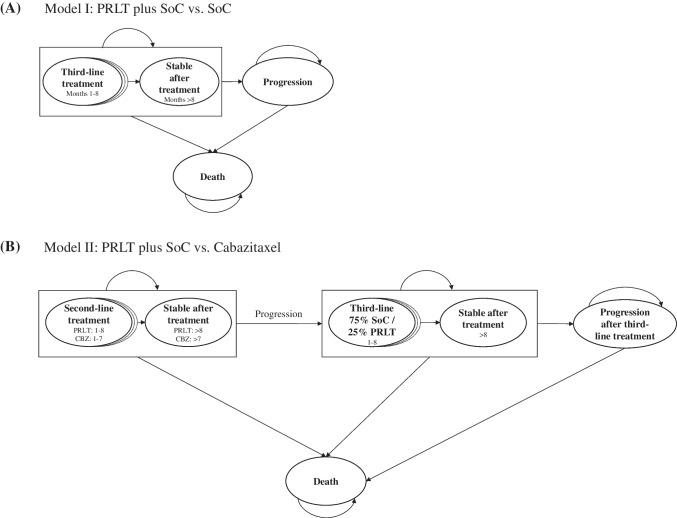
Model Structures **A**: Model Structures (Model I). Figure 1a depicts the structure of Model I. Each circle represents a health state, and each arrow depicts how patients may transition between health states. In each simulated treatment arm (PRLT plus SoC vs. SoC), treatment lasts for up to 8 months. Thereafter, all patients who have not progressed or died, move to the health state ‘stable after treatment’ where they remain until progression or death occurs. Changes between the states (i.e., cycle length) occur monthly. **B**: Model Structures (Model II). Figure 1b depicts the structure of Model II. Each circle represents a health state, and each arrow depicts how patients may transition between health states. In each simulated treatment arm (PRLT plus SoC vs. cabazitaxel), second-line treatment lasts for up to 8 or 7 months for PRLT or CBZ, respectively. Thereafter, all patients who have not progressed or died, move to the health state ‘stable after treatment’. After progression, 75% of the cohort is simulated to receive SoC treatment, and 25% of the cohort is simulated to receive PRLT for up to 8 months. If they do not progress or die during treatment, patients move after 8 months to the ‘stable after treatment’ state, where they remain until progression or death occurs. Changes between the states (i.e., cycle length) occur monthly. CBZ – cabazitaxel, PRLT – ^177^Lu-PSMA-617 radioligand therapy, SoC – Standard of Care

We developed two cohort discrete-time state-transition models to assess the cost-effectiveness of PRLT (Fig. 1). These models simulated a cohort of 1,000 patients who transitioned between the health states for each of the two treatment arms over time. Both models were time-inhomogeneous, meaning that the transition probabilities between health states were not constant but changed over time. The simulated cohort consisted of males who had advanced mCRPC and were PSMA-positive, eligible for treatment with PRLT, and received at least one androgen receptor pathway inhibitor and at least one taxane-based chemotherapy, similar to the pre-treatment characteristics of patients in the VISION (study population: *n* = 831) [5] and TheraP (study population: *n* = 200) trials [6].

Model I simulated the costs and effects of a cohort receiving PRLT plus SoC compared to a cohort receiving SoC alone as a third-line treatment for mCRPC (i.e., after at least two chemotherapy treatments). It consisted of four health states: (i) third-line treatment, (ii) stable after treatment, (iii) progression, and (iv) death. We defined PRLT as treatment with ^177^Lu-PSMA-617 radioligand therapy, and SoC as any supplementary individual treatment or active medication (e.g., androgen receptor pathway inhibitor). Model II simulated the costs and effects of PRLT plus SoC as a second-line treatment (i.e., after at least one chemotherapy treatment) compared to cabazitaxel. Following second-line treatment, 75% of the cohort was assumed to receive SoC, whereas 25% was assumed to receive PRLT plus SoC. This model consisted of six health states: (i) second-line treatment, (ii) stable after second-line treatment, (iii) third-line treatment after progression, (iv) stable after third-line treatment, (v) next progression, and (vi) death.

In line with previous research, we simulated 60 months as the cohorts’ lifetime for our model horizon [18, 19] and used a cycle length of one month. Tunnel states were incorporated to capture time-dependent costs associated with being under treatment and being stable. We assumed a treatment duration of eight months for both the PRLT plus SoC arm and for the SoC arm in Models I and II, and seven months for cabazitaxel. These durations were chosen conservatively to account for the maximum possible time under treatment. For example, PRLT was administered every six weeks for up to six cycles, corresponding to eight months. We applied the life table method as this is the recommended method for within-cycle correction to improve estimations [23, 24] and used a 3% discount rate for costs and effects in the base case as recommended for the German context [25]. We calculated ICERs by dividing the differences in costs and effects between PRLT plus SoC and the comparator. Model estimations were conducted using the heemod package for R [26]. The reporting of this study adheres to the CHEERS 2022 statement (Online Resource 1) [27].

### Model parameters

#### Transition probabilities

We derived dynamic transition probabilities between states by fitting accelerated failure time hazard models to published Kaplan Meier curves for progression-free survival (PFS) and overall survival (OS) from the VISION trial [28] (Model I) and the TheraP trial (Model II) [6, 7]. PFS determined the transitions between being under treatment/stable and progression, whereas OS determined transitions to the death state. We used the WebPlotDigitizer [29] and Guyot et al.’s method [29] to extract data from the published Kaplan–Meier curves. Following established guidelines for fitting and extrapolating hazard models [30, 31], we evaluated several parametric distributions (i.e., Weibull, exponential, gamma, log-normal, Gompertz, log-logistic and Generalized Gamma) for their fit based on visual inspection, the Akaike Information Criterion and the Bayesian Information Criterion (Online Resources 2/3). To improve the fit of the PFS hazard models for SoC (Model I) and cabazitaxel (Model II), we used piecewise models with knot values at 3.6 months for SoC and 4.5 months for cabazitaxel. Selected parametric distributions of the hazard models estimating the transition probabilities between states are summarized in Table 1. To explore the effect of extrapolation on the results, alternative specifications are reported in Online Resources 2/3.

**Table 1:** Parametric distributions for transition between states

Model	Treatment Strategy	Endpoint	Parametric Distribution	Base Case
I	PRLT	OS	Log-logistic	Shape: 1.9263, Scale: 15.5377
		PFS	Log-normal	Meanlog: 2.1048, SDlog: 0.9841
	SoC	OS	Gamma	Shape: 1.6065, Rate: 0.1140
		PFS, < 3.6 months	Log-logistic	Shape: 4.7332, Scale: 2.3643
		PFS, ≥ 3.6 months	Log-normal	Meanlog: 2.2763, SDlog: 0.8268
II	PRLT	OS	Log-logistic	Shape: 1.9576, Scale: 18.6763
		PFS	Log-normal	Meanlog: 1.5854, SDlog: 0.9737
	Cabazitaxel	OS	Log-logistic	Shape: 2.2526, Scale: 19.3004
		PFS, < 4.5 months	Log-normal	Meanlog: 0.4939, SDlog: 0.5765
		PFS, ≥ 4.5 months	Log-normal	Meanlog: 2.0563, SDlog: 0.2874
		*Subsequent treatment according to Model I*		

*OS* Overall survival, *PFS* Progression-free survival, *PRLT* ^177^Lu-PSMA-617 radioligand therapy, *SoC* Standard of Care

#### Costs derived from statutory health insurance claims data

To represent the perspective of the German statutory health insurance system, we derived costs from claims data for health services utilized between 1 January 2019 and 31 December 2022. These data, which included real-world costs for inpatient stays, outpatient physician contacts, outpatient drug prescriptions, medical supplies, assistive devices, and rehabilitative therapies, were obtained from a large, anonymized dataset provided by GWQ ServicePlus AG, a joint venture of medium-sized statutory health insurers. The dataset covered approximately 6.3 million individuals insured in Germany, representing about 6% of the German population covered by statutory health insurance. Because reimbursement practices are uniform across the German statutory health insurance system, the data reliably reflected the treatment received by the German statutory health insurance population, which comprises almost 90% of the population in Germany [32].

The sample comprised men with continuous insurance coverage during the observation period or until death who had claims with at least one inpatient or at least two outpatient prostate cancer diagnoses. As is standard in analyses using German claims data, confirmation of an outpatient diagnosis required a second outpatient diagnosis in a subsequent quarter [33]. Additionally, at least one diagnosis indicating metastases was required in the same quarter as the prostate cancer diagnosis. Based on previously established selection criteria for this cohort [16], patients with a history of other cancer diagnoses were excluded unless these represented comorbid neoplasms. Detailed criteria for patient identification in the claims data analysis can be found in Online Resource 4.

Individuals in this sample were assigned to different groups for cost estimation. Claims data from 77 individuals with at least two claims for PRLT within 90 days were used to estimate costs for the PRLT plus SoC treatment strategy; no claims for chemotherapy were observed in this group. For the SoC treatment strategy, cost estimates were based on claims data from 1,023 individuals who received abiraterone, enzalutamide, docetaxel, or cabazitaxel, with at least one claim for one of these drugs and no claims for PRLT. Lastly, claims data from 102 individuals with at least three claims for cabazitaxel within 90 days [16] were used to estimate costs for the cabazitaxel treatment strategy in Model II.

Costs for the ‘under treatment’ state were based on the initial 90-day observation period, whereas costs for ‘stable after treatment’ state were derived from the mean costs observed between 360 and 540 days after initial treatment (see Table 2 for input parameters).

**Table 2:** Input parameters for costs and utilities

Attribute/Model	Treatment Strategy	State	Base Case	Source	Deterministic Sensitivity Analysis		Probabilistic Sensitivity Analysis	
					Lowest Value	Highest Value		
***Costs [€]***							***Gamma*** ***Mean***	***SD***
I	PRLT	Third-line treatment	7,340	Claims data	3,670	11,010	7,340	2,942
		Stable after treatment	3,990	Claims data	1,995	5,985	3,990	3,099
		Progression	3,990	Claims data	1,995	5,985	3,990	3,099
		Death	0					
	SoC	Third-line treatment	6,716	Claims data	3,358	10,074	6,716	3,818
		Stable after treatment	4,794	Claims data	2,397	7,191	4,794	4,932
		Progression	4,794	Claims data	2,397	7,191	4,794	4,932
		Death	0					
II	PRLT	Second-line treatment	7,340	Claims data	3,670	11,010	7,340	2,942
		Stable after treatment	3,990	Claims data	1,995	5,985	3,990	3,099
		[25%] Third-line treatment with PRLT after first progression	7,340	Claims data	3,670	11,010	7,340	2,942
		Stable after third-line PRLT treatment	3,990	Claims data	1,995	5,985	3,990	3,099
		Next progression after third-line PRLT	3,990	Claims data	1,995	5,985	3,990	3,099
		[75%] Third-line treatment with SoC after first progression	6,716	Claims data	3,358	10,074	6,716	3,818
		Stable after third-line SoC treatment	4,794	Claims data	2,397	7,191	4,794	4,932
		Next progression after third-line SoC	4,794	Claims data	2,397	7,191	4,794	4,932
		Death	0					
	Cabazitaxel	Second-line treatment	14,460	Claims data	7,230	21,690	14,460	4,760
		Stable after treatment	8,123	Claims data	4,062	12,185	8,123	12,422
		[25%] Third-line treatment with PRLT after first progression	7,340	Claims data	3,670	11,010	7,340	2,942
		Stable after third-line PRLT treatment	3,990	Claims data	1,995	5,985	3,990	3,099
		Next progression after third-line PRLT	3,990	Claims data	1,995	5,985	3,990	3,099
		[75%] Third-line treatment with SoC after first progression	6,716	Claims data	3,358	10,074	6,716	3,818
		Stable after third-line SoC treatment	4,794	Claims data	2,397	7,191	4,794	4,932
		Next progression after third-line SoC	4,794	Claims data	2,397	7,191	4,794	4,932
		Death	0					
***Utilities***							***Beta*** ***Shape 1***	***Shape 2***
I	PRLT	Third-line treatment	0.7492	[34]	0.5619	0.9365	2.6590	0.8901
		Stable after treatment	0.7492	[34]	0.5619	0.9365	2.6590	0.8901
		Progression	0.6440	[34]	0.4830	0.8050	1.8359	1.0149
		Death	0.0					
	SoC	Third-line treatment	0.6946	[34]	0.5210	0.8683	1.7945	0.7890
		Stable after treatment	0.6946	[34]	0.5210	0.8683	1.7945	0.7890
		Progression	0.6460	[34]	0.4845	0.8075	1.5734	0.8622
		Death	0.0					
II	PRLT	Second-line treatment	0.7492	Assumption based on [34]	0.5619	0.9365	2.6590	0.8901
		Stable after treatment	0.7492	Assumption based on [34]	0.5619	0.9365	2.6590	0.8901
		[25%] Third-line treatment with PRLT after first progression	0.7492	[34]	0.5619	0.9365	2.6590	0.8901
		Stable after third-line PRLT treatment	0.7492	[34]	0.5619	0.9365	2.6590	0.8901
		Next progression after third-line PRLT	0.6440	[34]	0.4830	0.8050	1.8359	1.0149
		[75%] Third-line treatment with SoC after first progression	0.6946	[34]	0.5210	0.8683	1.7945	0.7890
		Stable after third-line SoC treatment	0.6946	[34]	0.5210	0.8683	1.7945	0.7890
		Next progression after third-line SoC	0.6460	[34]	0.4845	0.8075	1.5734	0.8622
		Death	0.0					
	Cabazitaxel	Second-line treatment	0.7260	[35]	0.5445	0.9075	1.2698	0.4792
		Stable after treatment	0.7260	[35]	0.5445	0.9075	1.2698	0.4792
		[25%] Third-line treatment with PRLT after first progression	0.7492	[34]	0.5619	0.9365	2.6590	0.8901
		Stable after third-line PRLT treatment	0.7492	[34]	0.5619	0.9365	2.6590	0.8901
		Next progression after third-line PRLT	0.6440	[34]	0.4830	0.8050	1.8359	1.0149
		[75%] Third-line treatment with SoC after first progression	0.6946	[34]	0.5210	0.8683	1.7945	0.7890
		Stable after third-line SoC treatment	0.6946	[34]	0.5210	0.8683	1.7945	0.7890
		Next progression after third-line SoC	0.6460	[34]	0.4845	0.8075	1.5734	0.8622
		Death	0.0					

*PRLT* ^177^Lu-PSMA-617 radioligand therapy, *SD* Standard deviation, *SoC* Standard of Care

#### Health-related quality of life derived from trial data

We used the EQ-5D-5L utilities from the VISION trial [34] to model health-related quality of life for PRLT plus SoC and SoC alone in Models I and II. Mean utilities were reported by treatment cycle; we averaged these to represent health-related quality of life for the ‘under treatment’ state. The utility reported at the end of treatment was used for the ‘progression’ state. For Model II, EQ-5D-5L utility data for cabazitaxel were similarly averaged over treatment cycles using data from the CARD trial [35] to model the ‘under treatment’ state. Due to limited evidence on health-related quality of life for second-line PRLT treatment, we assumed that utility values for second- and third-line treatment with PRLT matched those used in Model I (see Table 2 for input parameters).

### Sensitivity analyses and scenarios

We conducted deterministic and probabilistic sensitivity analyses to explore the robustness of the models to parameter changes (see Table 2 for input parameters). Deterministic one-way sensitivity analyses explored discount rates of 0% and 5%, longer time horizons of seven and 10 years, treatment costs varying by ± 50%, and utility values varying by ± 25%. For the probabilistic sensitivity analyses, we conducted a Monte Carlo simulation with 10,000 iterations, resampling the fitted hazard models and assuming a gamma distribution for costs and a beta distribution for utilities. Additionally, we calculated ICERs for alternative hazard model distributions (Online Resources 2/3) and for various scenarios (see Table 3 for scenario descriptions).

**Table 3:** Scenario analyses

Description	Adaption Before Implementation	Scenario Inputs	Results
Price of commercially available alternative according to German Benefit Assessment [36] instead of price for self-produced for PRLT (Model I, Model II)	Costs for single-dose vial normalized to claims data output, average 20% discount applied, added to mean inpatient costs*	Under (PRLT) treatment: €21,303	Model I: Incr. costs: €103,586 Incr. effect: 0.39 QALYs ICER: €264,367/QALY Model II: Incr. costs: €59,112 Incr. effect: 0.11 QALYs ICER: €550,686/QALY
Cabazitaxel cost data from older claims analysis of Kreis et al. [16] (Model II)	Updated to 2022 price levels, assumption of resource consumption under being stable: 55% of being under treatment	Under (cabazitaxel) treatment: €8,718; stable: €4,795	Incr. costs: €9,778 Incr. effect: 0.11 QALYs ICER: €91,095/QALY
If the cost data for the SoC cohort contained 59% of patients that received Radium-223 (share according to Kreis et al. [16])	Approximating the costs for receiving Radium-223 (annual therapy costs of Radium-223 [38] converted to monthly costs and adapted to 2022 price levels) and correcting costs for SoC accordingly	Under (SoC) treatment costs: 59% receiving Radium-223 at €6,716; 41% receiving any SoC treatment at €3,646 per month	Incr. costs: €30,418 Incr. effect: 0.39 QALYs ICER: €77,632/QALY
Variation of third-line treatment in Model II (base case: 75% SoC; 25% PRLT + Soc)		i) 25% SoC; 75% PRLT + SoC	i) Incr. costs: €−4,503 Incr. effect: 0.07 QALYs PRLT dominates cabazitaxel
		ii) 100% PRLT + SoC	ii) Incr. costs: €−6,024 Incr. effect: 0.06 QALYs PRLT dominates cabazitaxel
		iii) 100% SoC	iii) Incr. costs: €61 Incr. effect: 0.12 QALYs ICER: €490/QALY

* No correction for avoided costs of in-house production

*ICER* Incremental Cost Effectiveness Ratio, *PRLT* ^177^Lu-PSMA-617 radioligand therapy, *QALY* Quality-Adjusted Life Year, *SoC* Standard of Care

We developed several scenarios to account for varying assumptions about drug pricing, eligibility for Radium-223 in the SoC cost data, and the use of third-line therapy in Model II. Because PRLT was not commercially available during the cost observation period and treatments were based on patient-specific, in-house production under authorization-free use, one scenario assumed reimbursement at the proposed commercial price for PRLT minus an average rebate^2^ of 20% [36, 37]. Furthermore, we used published cabazitaxel cost data from Kreis et al. [16], which were derived from earlier claims data from a different statutory health insurance and applied different inclusion criteria. We also considered the possibility that metastasis patterns differed between the cohort from which SoC cost data were drawn and the cohort receiving PRLT. To account for this, we modeled a theoretical maximum in which up to 59% of the SoC cohort received Radium-223 [16], which is indicated exclusively for individuals with bone metastases. For third-line therapy in Model II, the scenarios were: (i) reversing the base case weights, with 75% of the cohort receiving PRLT plus SoC and 25% receiving SoC, (ii) 100% of the cohort receiving PRLT plus SoC, and (iii) 100% of the cohort receiving SoC.

## Results

The results of the cost-utility analyses are summarized in Table 4 for each model and treatment arm. Model I showed incremental costs of €27,200 per patient and an incremental gain of 0.39 QALYs per patient for PRLT plus SoC compared to SoC alone. The greater benefits of PRLT can be attributed to the cohort that received PRLT remaining longer in the ‘stable after treatment’ health state and, on average, progressing and dying later than the cohort that received SoC alone. After 60 months, approximately 96.3% of the cohort in this model had transitioned to the ‘death’ state. The ICER amounted to €69,418 per QALY gained.

**Table 4:** Results of cost-utility analyses

		Model I		Model II	
		PRLT plus SoC	SoC alone	PRLT plus SoC	Cabazitaxel
Base Case Analysis	Incurred costs (€)	97,732	70,532	98,303	99,763
	Incurred effect (QALY)	1.14	0.75	1.01	0.90
	Incremental costs (€)	27,200		−1,460	
	Incremental effect (QALY)	0.39		0.11	
	ICER (€ per QALY)	69,418		PRLT dominates cabazitaxel	
Probabilistic Sensitivity Analysis	Incurred costs (€)	106,640	71,106	98,786	100,227
	Incurred effect (QALY)	1.15	0.75	0.84	0.75
	Incremental costs (€)	35,534		−1,441	
	Incremental effect (QALY)	0.40		0.09	
	ICER (€ per QALY)	88,922		PRLT dominates cabazitaxel	

*PRLT* ^177^Lu-PSMA-617 radioligand therapy, *QALY* Quality-Adjusted Life Year, *SoC* Standard of Care

Model II suggested cost savings of €1,460 per patient and an incremental gain of 0.11 QALYs per patient for PRLT plus SoC compared to cabazitaxel as second-line therapy, indicating that PRLT plus SoC dominated cabazitaxel. After 60 months, 97.7% of the cohort in this model had transitioned to the ‘death’ state.

**Fig. 2:**
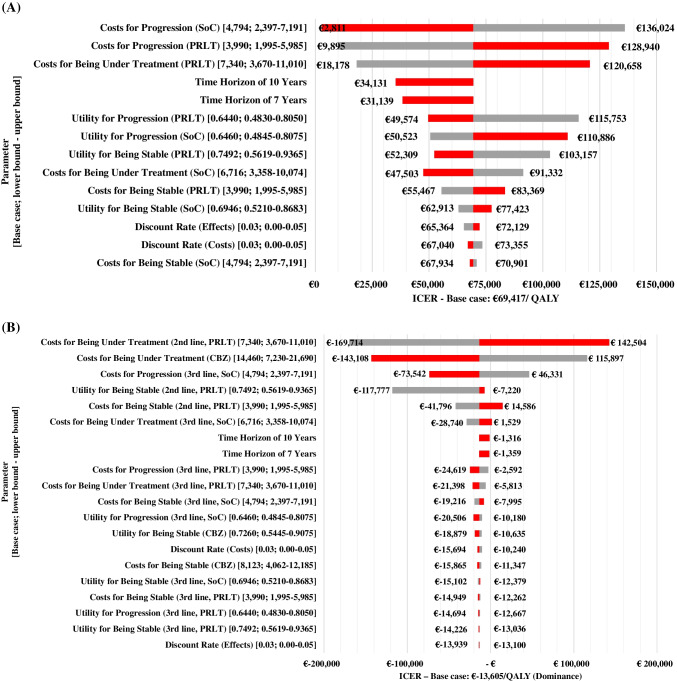
Tornado diagrams **A**: Tornado Diagrams (Model I) Fig. 2a shows the results of the one-way sensitivity analyses for Model I, i.e., each parameter was varied one at a time to assess the effect of parameter variation on the ICER. The base case value and the range for variation are given for each parameter on the left in brackets. The variation of the ICER is depicted next to the bars on the right. Grey (and red) bars show the result of the lower bound (and upper bound) variation. Parameters appear in the order of their influence on results from top to bottom **B**: Tornado Diagrams (Model II) Fig. 2b shows the results of the one-way sensitivity analyses for Model II, i.e., each parameter was varied one at a time to assess the effect of parameter variation on the ICER. The base case value and the ranges for variation are given on the left for each parameter. Variation in relation to the ICER result is depicted next to the bars on the right. Grey (and red) bars show the result of the lower bound (and upper bound) variation. Parameters appear in the order of their influence on results from top to bottom. CBZ – cabazitaxel, ICER – Incremental Cost-Effectiveness Ratio, PRLT – ^177^Lu-PSMA-617 radioligand therapy, SoC – Standard of Care, QALY – Quality-Adjusted Life Year

The ICER result of Model I was most sensitive to the costs in the ‘progression’ state of both SoC and PRLT plus SoC, followed by the treatment cost of PRLT plus SoC itself (Fig. 2). In contrast, the ICER result of Model II was most sensitive to the treatment costs of PRLT and cabazitaxel in the ‘second-line treatment’ state, followed by the costs in the ‘progression’ state after third-line SoC treatment.

To assess the uncertainty of our model, we calculated Monte-Carlo simulations with 10,000 iterations. For Model I, 66.7% of the iterations indicated higher costs with gains in QALYs for PRLT compared to SoC (probabilistic ICER for Model I: €88,922, Online Resource 5). For Model II, 79.7% of the iterations in the eastern quadrants of the cost-effectiveness plane indicated that PRLT was more effective than cabazitaxel while emphasizing uncertainty regarding the relative costs of PRLT (probabilistic result for Model II: PRLT dominated cabazitaxel, Online Resource 5).

## Discussion

This study provided cost-effectiveness estimates for treatment with ^177^Lu-PSMA-617 radioligand therapy (PRLT) in mCRPC patients, using real-world claims data from statutory health insurers in Germany. Previous modeling approaches have approximated clinical practice by dividing the comparator cohort between SoC (or best supportive care, depending on the definition) and cabazitaxel [20, 21]. In contrast, we simulated third-line mCRPC treatment by comparing PRLT to SoC, and second-line mCRPC treatment by comparing PRLT to cabazitaxel, thereby reflecting current clinical practice in Germany. According to the results of Model I, PRLT plus SoC led to a greater QALY gain but incurred higher costs than SoC alone. However, model-based estimates have suggested that the implicit willingness-to-pay threshold in Germany lies between €40,000 and €90,000 per QALY [39, 40]. A recent estimate for reimbursed cardiovascular drugs placed the threshold at €11,000 per life year gained [41]. The costs per QALY gained for PRLT were similar to that of other reimbursed precision oncology therapies in Germany, whose ICERs have ranged from approximately €15,000 to €30,000 [42, 43] to over €300,000 [44] per QALY. By these standards, PRLT plus SoC compared to SoC would likely be considered cost-effective.

According to the base case of Model II, PRLT was more effective and less costly than cabazitaxel. While this result is clearly in favor of PRLT, it is subject to high uncertainty. For instance, the data for OS and PFS for both treatment strategies were drawn from the Phase II TheraP trial, which was not powered to assess secondary outcomes like OS [6, 7]. Moreover, clinical practice is evolving rapidly, and the optimal treatment standard *after* PRLT is unclear [45]. Nevertheless, our scenario analysis suggests that the choice of subsequent treatment has only a minor impact on the results of Model II.

More importantly, as with Model I, the sensitivity analysis for Model II indicated that costs were highly influential. On the one hand, due to the period of claims data availability, PRLT treatment costs reflected reimbursement for the self-formulated drug produced in-house by hospital pharmacies or nuclear medicine departments. In late 2022, however, a commercially produced PRLT was granted marketing authorization in Europe, which is expected to be more expensive for health care providers than in-house production. To approximate the potential price effect, we assumed the use of a commercially produced PRLT, adapting the treatment price mentioned in the German benefit assessment minus the average rebate [36, 37]. This resulted in substantially higher ICERs for both models (> €250,000 per QALY gained, see Table 3 for scenario results). On the other hand, using the alternative cost data from Kreis et al., for which inflation-adjusted cabazitaxel treatment costs were approximately 40% lower than those in our sample [16], increased the ICER of Model II to about €90,000 per QALY gained. Under these conditions, the results no longer support the dominance of PRLT over cabazitaxel for second-line treatment. Nevertheless, we consider the claims data used in our base-case analysis to be more appropriate for capturing costs in patients with advanced mCRPC for two reasons: First, our stricter selection criteria probably identified mCRPC patients with a higher disease burden than those in the analysis by Kreis et al. Second, Kreis et al. did not provide cost data for PRLT, meaning that while their cost estimates for cabazitaxel reflect a patient population with a lower disease burden (due to different inclusion criteria), no corresponding adjustment to PRLT costs can be made.

### Limitations

Our analysis has several limitations. First, as described above, our results were highly influenced by the cost data inputs. Although costs aggregated from statutory health insurers reflect a real-life statutory health insurance perspective, the dataset did not include information on sick leave payments, which may have led to an underestimation of total incurred costs. However, this is likely of minor relevance given that mCRPC patients are predominantly older than working age [46], and sick leave payments were found to be minimal in a previous analysis [16].

Second, our cost-utility models relied on assumptions that may be subject to debate. We opted for time-inhomogeneous state-transition models instead of partitioned survival models, which are commonly used in advanced cancer modeling, to allow Model I to be nested in Model II as part of the treatment sequence. Nonetheless, the incremental QALY results for Model I appeared consistent with estimates for other countries using partitioned survival models [18, 19]. Reported differences in effect sizes were modest, ranging from 0.03 to 0.05 [18, 19], and may be attributed to differences in the parametric distributions used for hazard modelling, as well as differences in model types. However, the assumption of equal mortality risk from the ‘stable’ or ‘progression’ health states –an acknowledged oversimplification inherent in both state-transition and partitioned survival models [47] – remains a limitation and a common point of critique [48].

Third, evidence for innovative radiopharmaceutical therapies in advanced mCRPC is limited. As a result, we did not differentiate costs or health-related quality of life for PRLT between second-line and third-line treatment in Model II. Furthermore, the evidence on OS and PFS outcomes for PRLT compared with cabazitaxel is sparse, adding to the uncertainty of the results. Ongoing clinical trials are investigating the use of PRLT in treatment sequencing and combinations [49].

Finally, our analysis simplified the choice of treatments by comparing only PRLT plus SoC versus SoC alone (Model I) and PRLT versus cabazitaxel (Model II). Specialized treatment options available in clinical practice were outside of our model’s scope, such as olaparib for patients with BRCA1 and BRCA2 mutations, or other types of PRLT (e.g., ^225^Ac-PSMA) [3].

## Conclusion

PRLT is a novel treatment option for advanced mCRPC, offering benefits beyond extended survival. Although Germany does not have an explicit willingness-to-pay threshold that could be used to guide interpretation of the ICER estimated in Model I, our results place the ICER within the range observed for other reimbursed cancer therapies. This suggests that PRLT may be a cost-effective option for the treatment of mCRPC. However, it is important to note that our cost data were drawn from the period between 2019 and 2022, during which PRLT was mainly produced in-house by hospitals, resulting in lower costs than those associated with the currently available commercial formulation.

## Supplementary Information

Below is the link to the electronic supplementary material.

Supplementary file1 (PDF 545 KB)

## Data Availability

Published information on OS, PFS and quality of life can be found in the respective publications cited in the text. Aggregated claims data cannot be made accessible.

## Footnotes

1 In the VISION trial, SoC was defined by referring to medication other than active anticancer treatment.

2 AMNOG (German: *Arzneimittelmarktneuordnungsgesetz*) is a German law regulating pharmaceutical pricing in the German statutory health insurance system. It establishes a process for negotiating rebates on newly launched pharmaceuticals based on an assessment of their added benefit, innovation and market competition, ultimately determining the reimbursed price.
