# Supplementary material for: Cost-utility analysis of 177Lu-PSMA-617 radioligand therapy in second-line and third-line treatment for metastatic castration-resistant prostate cancer (mCRPC) in Germany
Source: Eur J Nucl Med Mol Imaging. 2025 May 8;52(12):4377–89. doi: 10.1007/s00259-025-07317-9 (PMC12491114; doi:10.1007/s00259-025-07317-9)
Supplement: Supplementary file 1 — Supplementary file1 (PDF 545 KB) [file 259_2025_7317_MOESM1_ESM.pdf]

Supplementary Material

Cost-Utility Analysis of ^{177}Lu-PSMA-617 Radioligand Therapy in Second-Line and Third-Line Treatment for Metastatic Castration-Resistant Prostate Cancer (mCRPC) in Germany

European Journal of Nuclear Medicine and Molecular Imaging

Carolin Brinkmann, M.Sc.[1]*, ORCID: 0000-0002-2550-0306*

carolin.brinkmann@uni-hamburg.de

Richard P. Baum, Prof. Dr.[2,3]

Tom Stargardt, Prof. Dr. [1]

1 Universität Hamburg, Hamburg Center for Health Economics, Hamburg, Germany

2 International Centers for Precision Oncology (ICPO), Wiesbaden, Germany

3 CURANOSTICUM Wiesbaden-Frankfurt at DKD Helios Klinik, Wiesbaden, Germany

Corresponding author:

Carolin Brinkmann

Hamburg Center for Health Economics, Universität Hamburg

Esplanade 36

20354 Hamburg

carolin.brinkmann@uni-hamburg.de

Online Resources

Online Resource 1: CHEERS Checklist

Topic	No.	Item	Location where item is reported
**Title**			
	1	Identify the study as an economic evaluation and specify the interventions being compared.	Title
**Abstract**			
	2	Provide a structured summary that highlights context, key methods, results, and alternative analyses.	Abstract
**Introduction**			
Background and objectives	3	Give the context for the study, the study question, and its practical relevance for decision making in policy or practice.	Introduction
**Methods**			
Health economic analysis plan	4	Indicate whether a health economic analysis plan was developed and where available.	No prior registration
Study population	5	Describe characteristics of the study population (such as age range, demographics, socioeconomic, or clinical characteristics).	Methods, Model Structure/ Costs Derived from German Claims Data
Setting and location	6	Provide relevant contextual information that may influence findings.	Methods, Costs Derived from German Claims Data/ Discussion
Comparators	7	Describe the interventions or strategies being compared and why chosen.	Introduction, Aim/ Methods, Model Structure
Perspective	8	State the perspective(s) adopted by the study and why chosen.	Introduction, Aim
Time horizon	9	State the time horizon for the study and why appropriate.	Methods, Model Structure
Discount rate	10	Report the discount rate(s) and reason chosen.	Methods, Model Structure
Selection of outcomes	11	Describe what outcomes were used as the measure(s) of benefit(s) and harm(s).	Methods, Model Parameters
Measurement of outcomes	12	Describe how outcomes used to capture benefit(s) and harm(s) were measured.	Methods, Model Parameters
Valuation of outcomes	13	Describe the population and methods used to measure and value outcomes.	Methods, Model Parameters
Measurement and valuation of resources and costs	14	Describe how costs were valued.	Methods, Costs Derived from German Claims Data
Currency, price date, and conversion	15	Report the dates of the estimated resource quantities and unit costs, plus the currency and year of conversion.	Methods, Costs Derived from German Claims Data
Rationale and description of model	16	If modeling is used, describe in detail and why used. Report if the model is publicly available and where it can be accessed.	Methods, Model Structure
Analytics and assumptions	17	Describe any methods for analysing or statistically transforming data, any extrapolation methods, and approaches for validating any model used.	Methods, Model Parameters/ Online Resource B/C
Characterising heterogeneity	18	Describe any methods used for estimating how the results of the study vary for subgroups.	Not applicable

Characterising distributional effects	19	Describe how impacts are distributed across different individuals or adjustments made to reflect priority populations.	Not applicable
Characterising uncertainty	20	Describe methods to characterise any sources of uncertainty in the analysis.	Methods, Sensitivity Analyses and Scenarios
Approach to engagement with patients and others affected by the study	21	Describe any approaches to engage patients or service recipients, the general public, communities, or stakeholders (such as clinicians or payers) in the design of the study.	Not reported
**Results**			
Study parameters	22	Report all analytic inputs (such as values, ranges, references) including uncertainty or distributional assumptions.	Tables 1-3
Summary of main results	23	Report the mean values for the main categories of costs and outcomes of interest and summarise them in the most appropriate overall measure.	Results, section 1-2, Table 4
Effect of uncertainty	24	Describe how uncertainty about analytic judgments, inputs, or projections affect findings. Report the effect of choice of discount rate and time horizon, if applicable.	Results, section 3-4, Figure 2-3
Effect of engagement with patients and others affected by the study	25	Report on any difference patient/service recipient, general public, community, or stakeholder involvement made to the approach or findings of the study	Not reported
**Discussion**			
Study findings, limitations, generalisability, and current knowledge	26	Report key findings, limitations, ethical or equity considerations not captured, and how these could affect patients, policy, or practice.	Discussion
**Other relevant information**			
Source of funding	27	Describe how the study was funded and any role of the funder in the identification, design, conduct, and reporting of the analysis	Funding statement
Conflicts of interest	28	Report authors conflicts of interest according to journal or International Committee of Medical Journal Editors requirements.	Conflict of interest statement

From: Husereau D, Drummond M, Augustovski F, et al. Consolidated Health Economic Evaluation Reporting Standards 2022 (CHEERS 2022) Explanation and Elaboration: A Report of the ISPOR CHEERS II Good Practices Task Force. *Value Health* 2022;25. doi:10.1016/j.jval.2021.10.008

Online Resource 2: Fit of Distributions for Hazard Models (Model I)

**Model**	**Treatment Strategy**	**Endpoint**	**Parametric Distribution**	**AIC**	**BIC**	**ICER, if used (€ per QALY)**
I	PRLT	OS	Weibull	2,750.9	2,759.5	67,259
			Exponential	2,815.7	2,820.0	66,792
			Gamma	2,745.2	2,753.8	68,038
			Lognormal	2,752.9	2,761.5	70,542
			Gompertz	2,781.2	2,789.9	64,492
			**Loglogistic**	**2,741.2**	**2,749.8**	**69,418**
			Generalized Gamma	2,745.6	2,758.6	68,567
		PFS	Weibull	1,803.3	1,811.2	70,275
			Exponential	1,824.8	1,828.8	67,339
			Gamma	1,797.6	1,805.5	70,443
			**Lognormal**	**1,785.2**	**1,793.1**	**69,418**
			Gompertz	1,818.3	1,826.2	69,142
			Loglogistic	1,794.3	1,802.3	69,568
			Generalized Gamma	1,787.2	1,799.0	69,398
	SoC	OS	Weibull	1,378.0	1,385.3	69,982
			Exponential	1,398.7	1,402.4	67,347
			**Gamma**	**1,376.4**	**1,383.6**	**69,418**
			Lognormal	1,381.8	1,389.1	64,379
			Gompertz	1,386.0	1,393.2	71,165
			Loglogistic	1,381.0	1,388.2	63,527
			Generalized Gamma	1,377.8	1,388.7	68,563
		PFS, <3.6 months	Weibull	225.1	231.0	69,821
			Exponential	332.7	335.7	67,627
			Gamma	231.4	237.3	69,306
			Lognormal	246.4	252.3	68,753
			Gompertz	234.5	240.5	70,046
			**Loglogistic**	**223.0**	**229.0**	**69,418**
			Generalized Gamma	226.8	235.7	69,775
		PFS, ≥3.6 months	Weibull	183.0	186.8	69,400
			Exponential	183.8	185.8	69,436
			Gamma	180.5	184.4	69,393
			**Lognormal**	**172.9**	**176.7**	**69,418**
			Gompertz	185.8	189.7	69,444
			Loglogistic	175.5	179.4	69,418
			Generalized Gamma	141.4	147.2	fit not suitable for extrapolation

Online Resource 3: Fit of Distributions for Hazard Models (Model II)

**Model**	**Treatment Strategy**	**Endpoint**	**Parametric Distribution**	**AIC**	**BIC**	**ICER**
II	PRLT	OS	Weibull	564.6	569.8	-21893
			Exponential	572.5	575.1	-238995
			Gamma	561.6	566.8	-11014
			Lognormal	557.2	562.4	-10064
			Gompertz	572.2	577.4	-94850
			**Loglogistic**	**556.5**	**561.7**	**-13605**
			Generalized Gamma	559.2	567.0	-10068
		PFS	Weibull	525.6	530.8	-7148
			Exponential	527.7	530.3	-19206
			Gamma	523.8	529.0	-6146
			**Lognormal**	**520.1**	**525.2**	**-13605**
			Gompertz	528.9	534.1	-13692
			Loglogistic	521.4	526.6	-12452
			Generalized Gamma	521.6	529.4	-12115
	Cabazitaxel	OS	Weibull	506.3	511.5	-5943
			Exponential	525.2	527.8	32712
			Gamma	505.0	510.2	-11257
			Lognormal	506.6	511.9	-16349
			Gompertz	513.4	518.6	13239
			**Loglogistic**	**505.0**	**510.2**	**-13605**
			Generalized Gamma	506.9	514.7	-12696
		PFS, <4.5 months	Weibull	134.6	138.7	-8473
			Exponential	160.1	162.1	-45980
			Gamma	132.7	136.8	-9149
			**Lognormal**	**131.1**	**135.2**	**-13605**
			Gompertz	140.2	144.3	-7584
			Loglogistic	136.0	140.1	-21182
			Generalized Gamma	no fit	no fit	no fit
		PFS, ≥4.5 months	Weibull	200.9	204.5	
			Exponential	263.8	265.6	-17812
			Gamma	194.1	197.6	-13725
			**Lognormal**	**192.5**	**196.0**	**-13605**
			Gompertz	213.4	217.0	
			Loglogistic	194.9	198.4	-13652
			Generalized Gamma	193.5	198.8	-13469

Online Resource 4: Specifications for Claims Data Analysis

Used coding systems:

- International Statistical Classification of Diseases and Related Health Problems, 10th revision, German Modification (ICD-10-GM)
- German Anatomical Therapeutic Chemical (ATC)-Classification
- German procedure classification (“Operationen- und Prozedurenschlüssel”, OPS)
- German Diagnosis Related Groups catalogues (G-DRG)

Cohort	Selection criteria
**Individuals receiving PRLT**	• Gender = Male<br>• AND continuous insurance period since 01/01/2019 either until 31/12/2022 or until death<br>• AND (between 01/01/2019 and 31/12/2022) 1 x diagnosis C61 inpatient or outpatient treatment in hospital OR between 01/01/2019and 31/12/2022 2 outpatient diagnoses C61<br>• AND (between 01/01/2019 and 31/12/2022) no other C diagnoses (outpatient, inpatient, outpatient treatment in hospital) except C20, C41, C43, C44, C61, C67, C68, C77, C78, C79, C80, C85<br>• AND at least one diagnosis C77 OR C78 OR C79 in the same quarter as a diagnosis C61 (outpatient, inpatient, outpatient treatment in hospital)<br>• AND at least 2x claims DRG M10B OR OPS 8-530.d OR ATC V10XX05 within 90 days<br><br>Observation from date:<br>1st claim DRG M10B OR OPS 8-530.d OR ATC V10XX05
**Individuals receiving SoC**	• Gender = Male<br>• AND continuous insurance period since 01/01/2019 either until 31/12/2022 or until death<br>• AND (between 01/01/2019 and 31/12/2022) 1 x diagnosis C61 inpatient or outpatient treatment in hospital OR between 01/01/2019 and 31/12/2022 2 outpatient diagnoses C61<br>• AND (between 01/01/2019 and 31/12/2022) no other C diagnoses (outpatient, inpatient, outpatient treatment in hospital) except C20, C41, C43, C44, C61, C67, C68, C77, C78, C79, C80, C85<br>• AND at least one diagnosis C77 OR C78 OR C79 in the same quarter as a diagnosis C61 (outpatient, inpatient, outpatient treatment in hospital)<br>• AND (between 01/01/2019 and 31/12/2022) NO claim DRG M10B OR NO OPS 8-530.d OR NO ATC V10XX05<br><br>Observation from date<br>1st claim ATC L02BB04 OR ATC L02BX03 OR ATC L01CD04 OR ATC L01CD02 OR OPS 6-007.6 OR OPS 6-006.2 OR 6-006.1 OR 6-002.h
**Individuals receiving cabazitaxel**	• Gender = Male<br>• AND continuous insurance period since 01/01/2019 either until 31/12/2022 or until death

	• AND (between 01/01/2019 and 31/12/2022) 1 x diagnosis C61 inpatient or outpatient treatment in hospital OR between 01/01/2019 and 31/12/2022 2 outpatient diagnoses C61<br>• AND (between 01/01/2019 and 31/12/2022) no other C diagnoses (outpatient, inpatient, outpatient treatment in hospital) except C20, C41, C43, C44, C61, C67, C68, C77, C78, C79, C80, C85<br>• AND at least one diagnosis C77 OR C78 OR C79 in the same quarter as a diagnosis C61 (outpatient, inpatient, outpatient treatment in hospital)<br>• AND (between 01/01/2019 and 31/12/2022) NO claim DRG M10B OR NO OPS 8-530.d OR NO ATC V10XX05<br>• AND at least 3x ATC L01CD04 or OPS 6-006.1 in 90 days<br><br>Observation from date<br>1st claim ATC L01CD04 or OPS 6-006.1

Online Resource 5:

Fig 3: Probabilistic Sensitivity Analyses

Fig 3a*: Probabilistic Sensitivity Analyses (Model I)*

Fig 3a shows the results of the probabilistic sensitivity analysis using a Monte-Carlo simulation (n=10,000 repetitions) for Model II. Each repetition randomly chose a set of parameters considering the parameter distributions and calculates the ICER (grey dots). The dark dot shows the base case result. The percentages on the outside give the number of results by quadrant, i.e., 6,670 of 10,000 simulations were in quadrant I (gain in QALYs but more expensive). Quadrant II: 1,920/10,000; Quadrant III: 335/10,000; Quadrant IV: 1,075/10,000

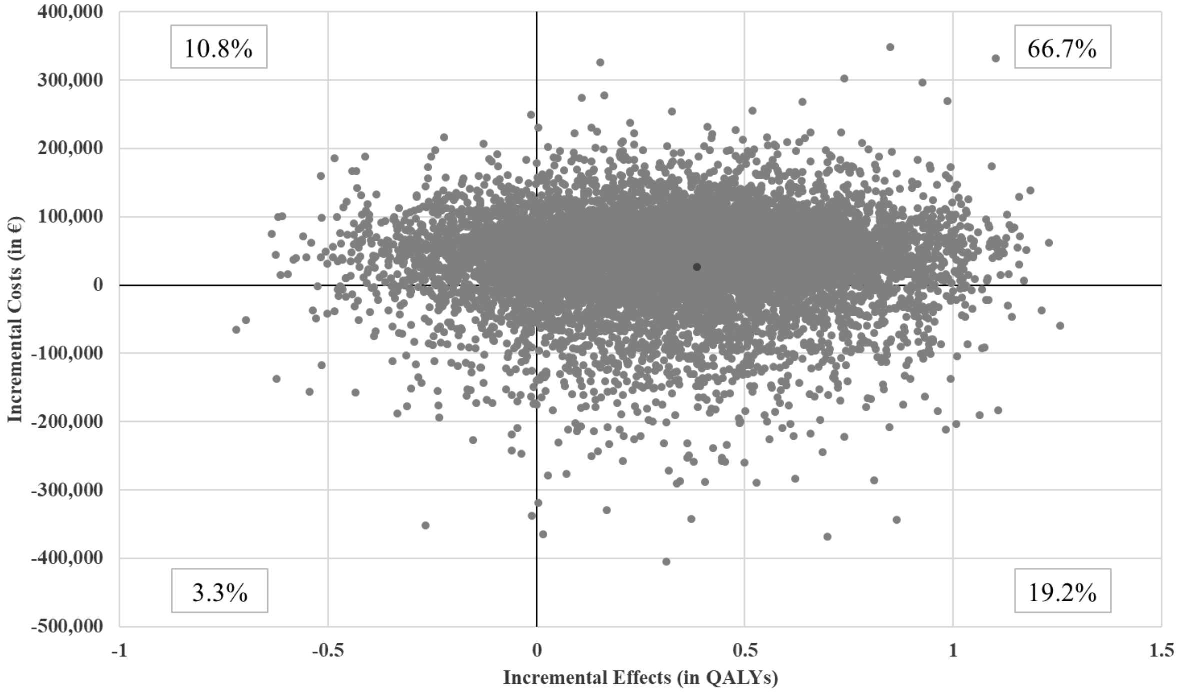

***Fig 3b**: Probabilistic Sensitivity Analyses (Model II)*

Fig 3b shows the results of the probabilistic sensitivity analysis using a Monte-Carlo simulation (n=10,000 repetitions) for Model II. Each repetition randomly chose a set of parameters considering the parameter distributions and calculates the ICER (grey dots). The dark dot shows the base case result. The percentages on the outside give the number of results by quadrant, i.e., 3,871 of 10,000 simulations were in quadrant I (gain in QALYs but more expensive). Quadrant II: 4,102/10,000, Quadrant III: 991 /10,000, Quadrant IV: 1,036 /10,000

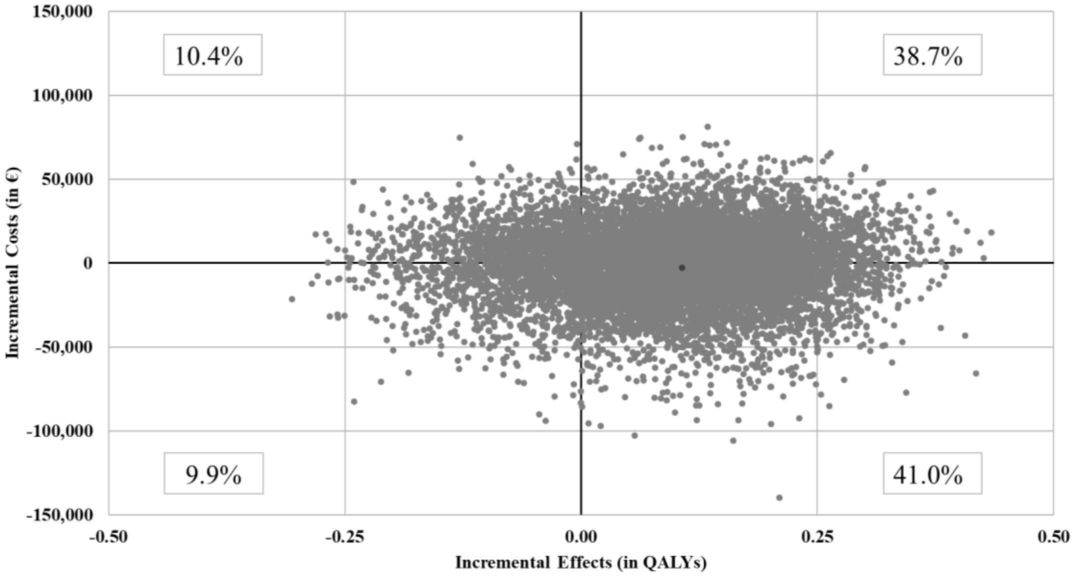

QALYs – Quality-Adjusted Life Years
